# Rethinking Cryptophane‑A for Methane Gas Sensing: Cross-Sensitivity to N_2_ and CO_2_ at Ambient Conditions

**DOI:** 10.1021/acs.analchem.5c03869

**Published:** 2025-09-30

**Authors:** Sebastián Alberti, Thierry Brotin, Jana Jágerská

**Affiliations:** † Department of Physics and Technology, UiT The Arctic University of Norway, NO-9037 Tromsø, Norway; ‡ Université Lyon 1, ENS de Lyon, CNRS UMR 5182, Laboratoire de Chimie, F-69364 Lyon, France

## Abstract

Since the affinity of Cryptophane-A for methane was first reported in 1993, cryptophane-doped polymer films have been extensively studied as enrichment cladding layers in plasmonic, fiber-optic, and integrated waveguide-based optical sensors. While the use of cryptophane-doped layers has improved methane sensitivity compared to undoped claddings, controversy has grown over the years regarding their claimed selectivity and practical applicability. In this work, we employ Raman spectroscopy to provide direct and unambiguous evidence that Cryptophane-A exhibits measurable affinity for three major atmospheric gases (carbon dioxide, methane, and nitrogen) at room temperature, debunking old beliefs of cryptophane-methane selectivity and reformulating the role of nitrogen. Notably, carbon dioxide shows a 1.5-times stronger affinity for Cryptophane-A than methane, while nitrogen relative affinity to methane was demonstrated to be 0.4. This study underscores the value of Raman spectroscopy as a benchmark technique for investigating gas capture within host molecules at ambient conditions. It offers deeper insight into the binding behavior of Cryptophane-A and enables quantification of its relative affinities to atmospheric gases, thereby revealing both the limitations and the potential of cryptophane for future sensing applications.

## Graphical abstract

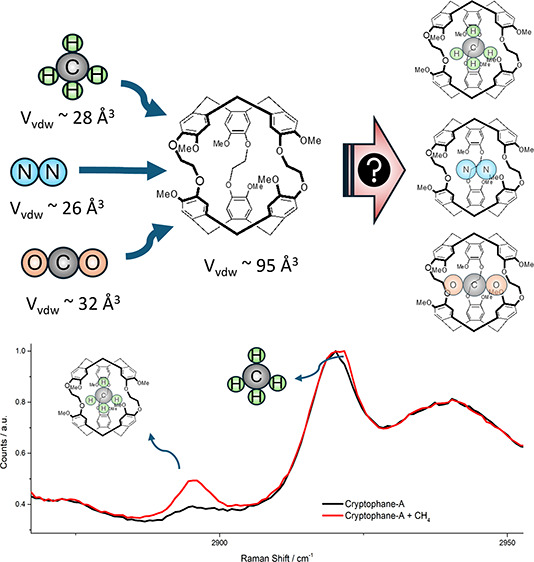

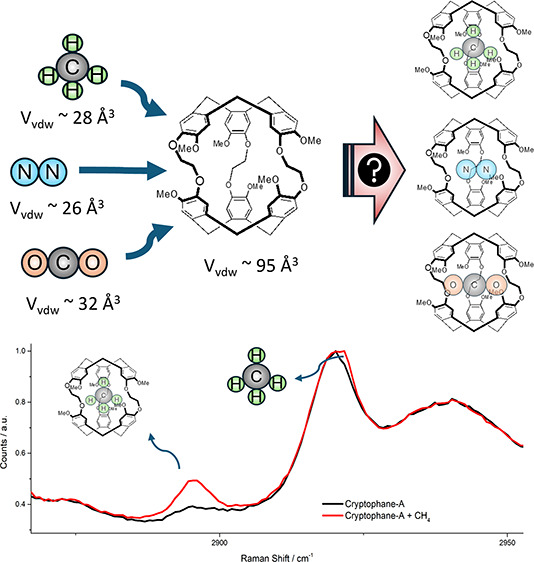

## Introduction

Cryptophanes, a class of hollow organic molecules with a well-defined internal cavities, have attracted attention for their ability to trap small molecules,^1,2^ ions,^3^ and even weakly interacting gases.^4,5^ For instance, the complexation of methane has inspired the development of cryptophane-doped cladding materials for methane sensing. When applied to nonspecific sensors such as optical fiber gratings,^6^ photonic crystals,^7^ Mach–Zehnder interferometers (MZI),^8^ surface plasmon resonance (SPR),^9^ or quartz crystal microbalances (QCM) sensor,^10^ these claddings have shown a significant methane enrichment, and, consequently, enhanced sensitivity compared to unclad sensors or polymer claddings without cryptophanes. Despite this progress, growing evidence from recent studies points to the intricate and sometimes problematic behavior of cryptophanes in practical sensing applications.^9^ One key issue is the conformational change, which can reduce the amount of active cryptophane sites, or generate new moieties that need to be evaluated. These structural alterations can decrease the availability of cryptophanes for guest binding, thereby diminishing the enrichment capability of the cladding and its practical utility. Conformational changes, such as imploded cryptophanes,^11^ were suggested decades ago^12^ and have recently been confirmed experimentally.^13^ Even more concerning is the growing number of reported interferences or competing guest molecules. Substances such as halomethanes, water,^14^ ethanol,^9^ nitrous oxide,^10^ ammonia^15^ and even hydrogen^16^ have all been identified as potential interferents, and new ones are continually being added. This increasing list raises questions about the assumed selectivity/specificity of cryptophane-doped claddings.

Interferences are key detrimental factors in nonspecific sensing setups, such as refractive index sensors, which are used in most studies involving cryptophane-doped claddings. In these systems, changes in temperature, pressure, humidity, or general adsorption of analytes to the polymer matrix—rather than to the cryptophane itself—can produce a measurable signal. As well, physical changes in the polymer, such as aging or swelling due to humidity, can further confound the measurements. While reference sensors, e.g., identical devices without cryptophanes in the cladding, can help mitigate these effects, inconsistent results from both optical refractive index sensor (e.g., MZI, SPR) and other nonspecific sensors such as QCM^10^ or Contact Potential Difference (CPD)^17^ sensors suggest that cryptophane cavity itself may be subject to interference from atmospheric gases beyond methane.

Traditionally, Proton Nuclear Magnetic Resonance (^1^H-NMR) spectroscopy has been the benchmark technique for studying methane-cryptophane binding.^1,16^ However, this approach is constrained by the requirement for low temperatures to resolve the ^1^H-NMR signal of the methane–cryptophane complex, necessitating extrapolation of the thermodynamic parameters to ambient conditions. Additionally, alternative guest molecules pose further complications. For example, H_2_ complexes are considered non detectable in normal operation conditions, N_2_ and CO_2_ lack hydrogen atoms, and long T_1_ relaxation times hinder ^13^C-NMR detection. As a result, interactions such as N_2_ with cryptophane-000 have only been inferred indirectly,^18^ while evidence for CO_2_-cryptophane-111 binding relies on subtle host signal splitting observed at cryogenic temperatures.^16^

In this work, we introduce Raman spectroscopy as a practical and complementary alternative to NMR spectroscopy. Unlike NMR, Raman spectroscopy does not depend on the presence of hydrogen and can identify and quantify aprotic gases such as N_2_ and CO_2_. We demonstrate that Raman spectra of CH_4_, N_2_, and CO_2_ exhibit distinct shifts when these gases are encapsulated in cryptophane-A compared to their free molecular forms (Figure 1). This enables the study of host–guest complex formation, and evaluation of selectivity to the selected cryptophane. In our approach, Cryptophane-A crystals and a commercial Raman microscope were used at room temperature, highlighting the simplicity yet effectiveness of the method.

**Figure 1:**
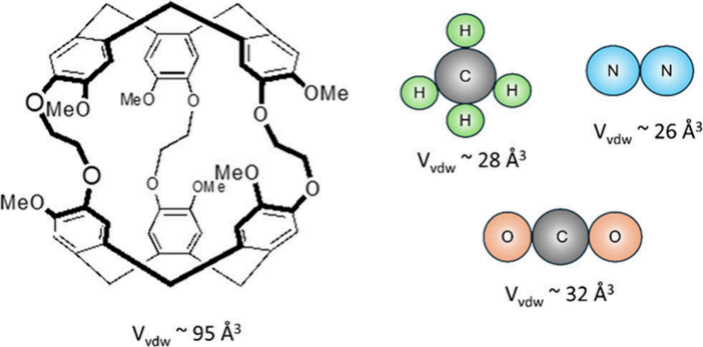
Schematic structure of Cryptophane-A, nitrogen, carbon dioxide, and methane. The Van der Waals volumes (V_vdw_) of the Cryptophane-A cavity and the respective gas molecules are also shown.

## Experimental Section

Cryptophane-A was synthesized with high purity (greater than 99%) using a multistep procedure previously reported by Brotin et al.^19^ (see Supporting Information, S1 for NMR data). The resulting crystals were placed in a custom-built flow chamber designed to interface with a Renishaw InVia Raman microscope. This chamber consisted of a glass slide with inlet and outlet openings (coupled to peek tubing), covered with a ∼500 um thick PDMS gasket and sealed on the top by a microscope cover glass 1.5 (160–190 um) (see Figure 2). The chamber construction facilitates the use of a 0.75 NA objective with tight focus, which provides a strong signal from a small active volume and can be precisely located over the cryptophane crystals, thus avoiding spurious signal from the coverslip or the chamber itself. The chamber inlet and outlet from the bottom of the microscope slide allowed us to introduce the gases of interest one per time and record the Raman spectra under controlled gas atmosphere. The measurements were performed under constant flow after signal stabilization, in successive measurements with 2 h in between to allow for elimination of any memory effects, such as from gases partitioned into the PDMS gasket material.

**Figure 2:**
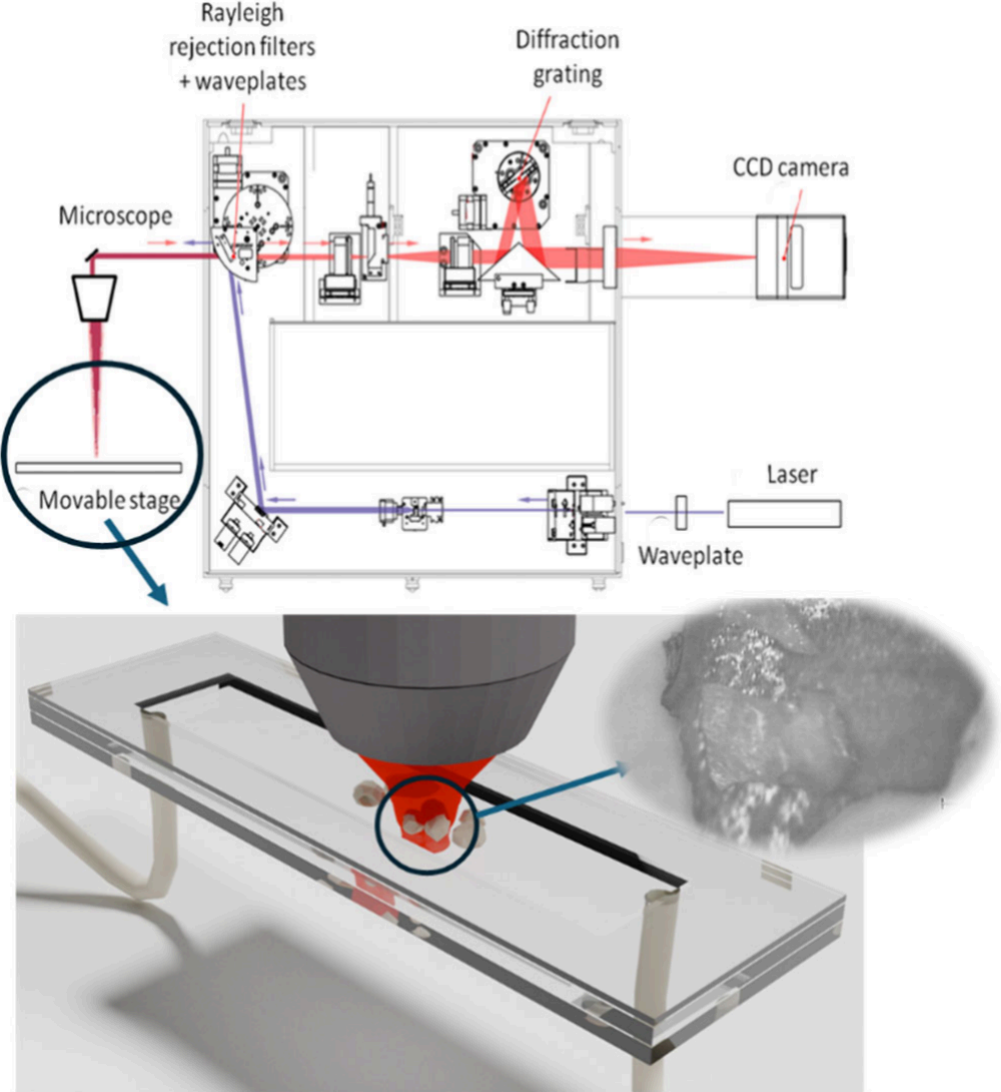
Schematic of the custom-built flow cell placed under confocal Raman microscope (Renishaw InVia). The diagram illustrates that the active volume includes both the solid crystal and the surrounding gas. Inset: image of the crystals taken with a 10X objective.

The spectrometer was configured to target specific Raman lines characteristic of the respective gases: 2917 cm^–1^ for methane, 1388 cm^–1^ for carbon dioxide, and 2329 cm^–1^ for nitrogen.^20^ These lines correspond to strong symmetric vibrational modes that produce intense Raman signals due to large changes in polarizability. A 785 nm laser was used for excitation, and each spectrum was obtained by averaging 200 measurements with 2-s integration time per measurement.

## Results and Discussion

An example of Raman spectra of Cryptophane-A under N_2_, CO_2_, and CH_4_ atmospheres is presented in Figure 3. For each gas, two distinct Raman peaks are observed superimposed on the background signal of Cryptophane-A. The right peak corresponds to the characteristic vibrational modes of the free gas, as reported in the literature: 2329 cm^–1^ for N_2_, 1388 cm^–1^ for CO_2_, and 2917 cm^–1^ for CH_4_.^20^ The second, noticeably red-shifted peak (i.e., at a lower wavenumber) is attributed to gas molecules encapsulated within the Cryptophane-A host structure. This red shift implies that the trapped molecules experience a different local environment, supporting the hypothesis of host–guest interactions within the cavity.

**Figure 3:**
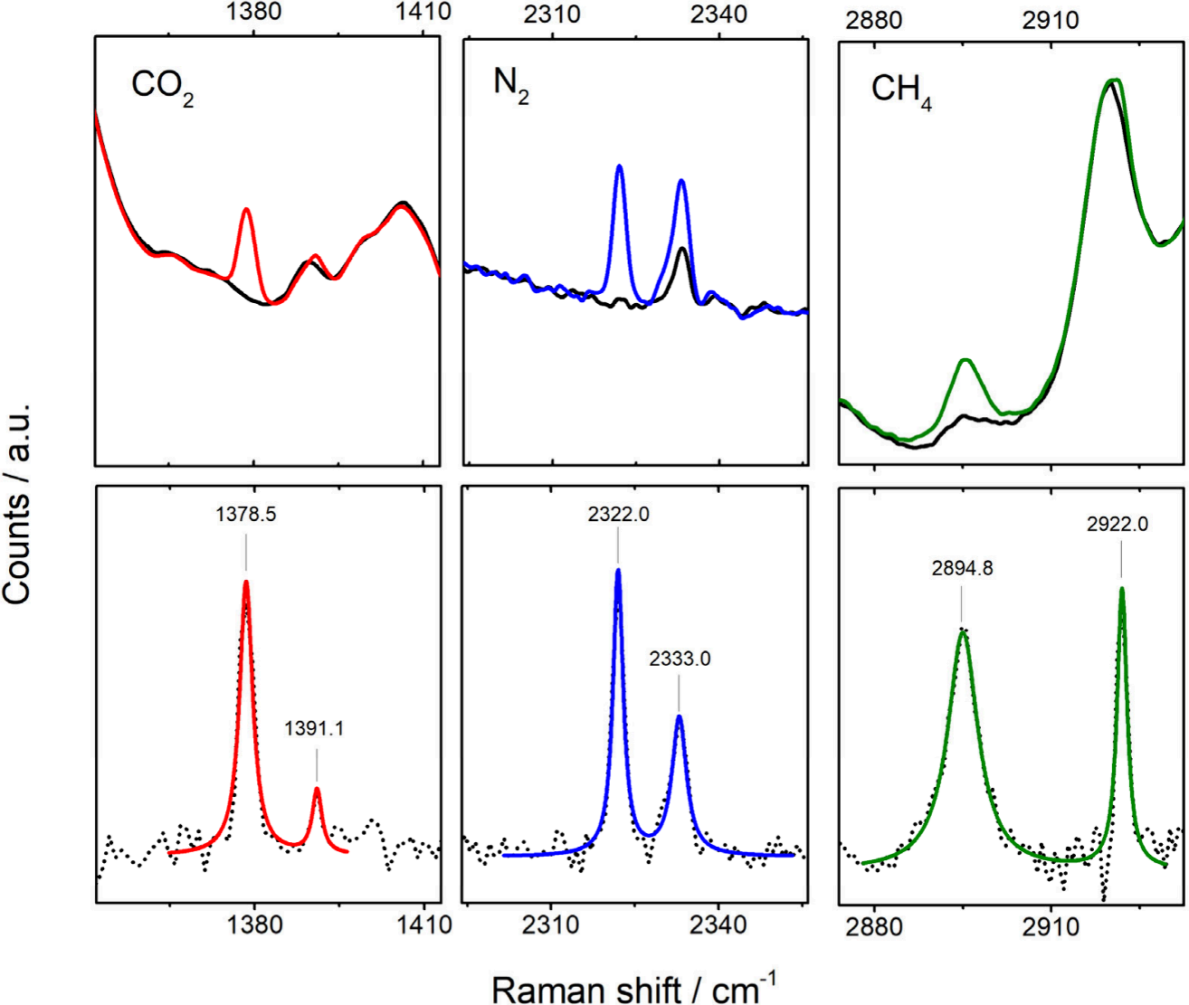
Top row: Raman spectra for CO_2_, CH_4_, and N_2_ recorded while focusing on a Cryptophane-A crystal, before (black) and after exposure to the respective gas (color). Background spectra (black) were collected when the gas chamber was purged with an alternative gas exhibiting no Raman features in the relevant spectral range. Bottom row: Background-subtracted spectra (shown as dotted lines) fitted with double Lorentzian functions (solid color lines).

The presence of two peaks indicates the coexistence of free and encapsulated gas within the Raman-active volume, i.e. the volume defined by the confocal volume of the objective lens equal to approximately 6.5 μm^3^. Since this volume exceeds the size of the Cryptophane-A crystals, some interaction with free gas is inevitable. To enhance detection of the encapsulated species, the focus was carefully adjusted to maximize the intensity of the red-shifted peaks. Following alignment, spectral measurements for CO_2_, N_2_, and CH_4_ were conducted without altering the focal plane or repositioning the sample. Maintaining a consistent overlap between the free gas and solid-phase volumes was essential for comparing the relative affinities of the three gases. To improve statistical robustness, measurements were repeated at two additional locations on the sample, each characterized by slightly different gas/solid overlap conditions.

For spectral analysis, background-corrected spectra were fitted using a double Lorentzian function, as shown in Figure 3. The obtained peak positions, their full width at half-maximum (fwhm), and the Raman red shifts are presented in Table S2.1 in Supporting Information, S2 for three sample locations (denoted as (1), (2), and (3)). Integrated peak intensities are analyzed in Figure 4.

**Figure 4:**
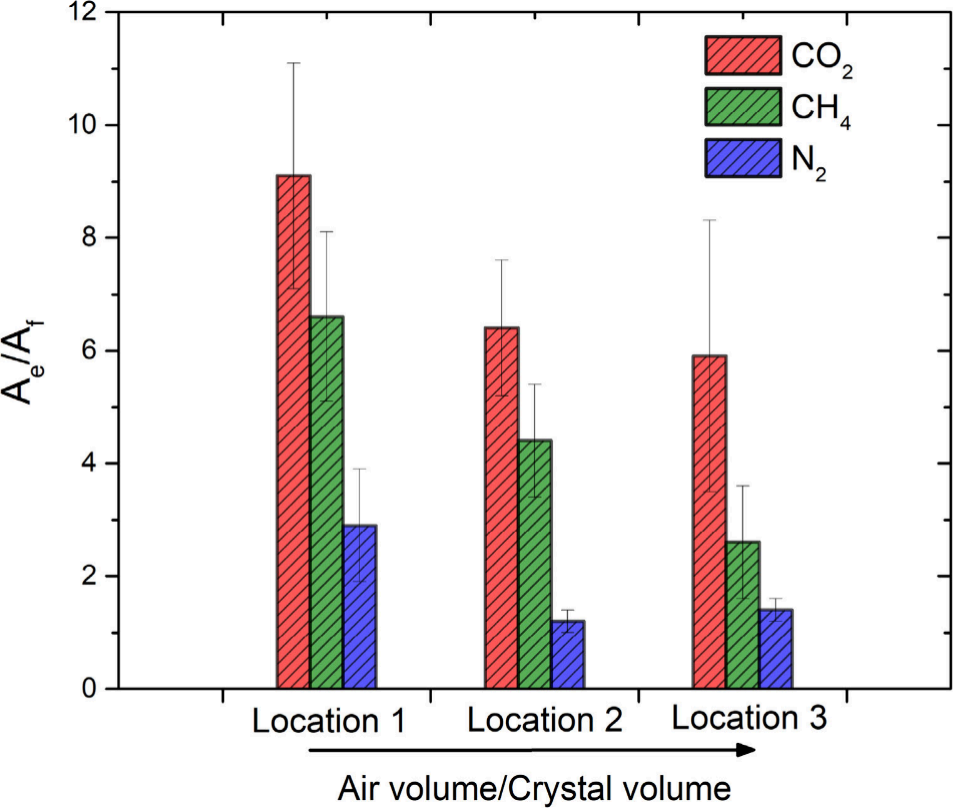
Bar graphic showing the relative area of bound gases (A_e_) normalized by the free gas (A_f_). The decrease in the magnitude implies a weaker interaction. Each group corresponds to a different focus position illuminating different crystals; therefore, the decrease in the amplitude between groups accounts for different air/crystal volumes.

All three gases, CO_2_, CH_4_, and N_2_, exhibit strong Raman signals in their encapsulated states, indicating that each can form a host–guest complex with Cryptophane-A. This observation suggests that competitive binding between gases is likely and challenges the notion of methane-selective sensing by Cryptophane-A-based sensors.

To estimate selectivity, the integrated intensities of the Raman peaks—i.e., areas under the peaks—were determined for both free (*A*_*f*_) and encapsulated (*A*_*e*_) gas. The resulting *A*_*e*_/*A*_*f*_ ratios are plotted in Figure 4 for each gas and location. While these ratios reflect the relative amounts of free and encapsulated gas within the Raman-active volume (assuming similar Raman cross sections for bound and free states), they do not directly yield enrichment due to uncertainties in gas–solid overlap. However, since the overlap was kept constant within each measurement location, comparing *A*_*e*_/*A*_*f*_ values between gases provides insight into the relative affinity of Cryptophane-A for each gas. This relative affinity, or selectivity, defined as the ratio between the binding constants (K_gas 1_/K_gas 2_) between gas pairs was calculated using the following expression:

$$S=\frac{K_{gas\;1}}{K_{gas\;2}}=\frac{{\left(A_{e}/A_{f}\right)}_{gas\;1}}{{\left(A_{e}/A_{f}\right)}_{gas\;2}}$$

The resulting selectivity values for CO_2_/CH_4_, CO_2_/N_2_, and CH_4_/N_2_, are listed in Table 1 for all three sample locations. While subject to uncertainties due to signal-to-noise ratios and limited spectral resolution, the average selectivity CO_2_/CH_4_ = 1.5, CO_2_/N_2_ = 4.5, and CH_4_/N_2_ = 2.5 clearly indicate competitive binding among the gases, with a preference of Cryptophane-A for CO_2_ > CH_4_ > N_2_.

**Table 1:** Selectivity of Cryptophane-A for CO_2_/CH_4_/N_2_

Gases	Location (1)	Location (2)	Location (3)	Average
CO_2_/CH_4_	1.5 ± 0.4	1.4 ± 0.4	2.2 ± 1.2	1.5 ± 0.3
CO_2_/N_2_	5.5 ± 1.3	3.2 ± 1.8	4.2 ± 1.8	4.5 ± 0.9
CH_4_/N_2_	3.7 ± 1.0	2.3 ± 0.9	1.9 ± 0.8	2.5 ± 0.5

The binding preference correlates with the gases’ boiling points (N_2_: −195 °C < CH_4_: −161 °C < CO_2_: −78 °C) and their corresponding polarizabilities (N_2_: 1.7 Å^3^, CH_4_: 2.6 Å^3^, CO_2_: 2.9 Å^3^).^21^ Higher polarizability generally enhances van der Waals interactions and the ability to induce dipoles in the surrounding environment, such as within the Cryptophane-A cavity. Similar effects have been observed in earlier studies comparing binding of CHCl_3_ (8.129 Å^3^) and CHF_2_Cl (4.440 Å^3^).^21^ The binding preference also increases according to molecular volume (N_2_: 26 Å^3^, CH_4_: 28 Å^3^, CO_2_: 32 Å^3^), although the differences between these three molecules are small, and all three molecules have a much smaller volume than other guests known for strong binding with cryptophane-A like chloroform (72 Å^3^) or xenon (42 Å^3^). While polarizability and size are often correlated, we expect molecular size to play a lesser role in this system.

In addition to integrated peak intensities, spectral broadening and red shifts of the Raman peaks offer further insight into host–guest interactions. The trapped methane exhibits particularly strong broadening compared to its free-state spectrum, which is consistent with confinement effects such as collisional broadening, as reported for methane clathrates.^22^ Although broadening is often related to the ratio of cavity size to molecular size, other factors — including molecular symmetry and relaxation/dephasing mechanisms — also contribute. For instance, despite its larger size, encapsulated CO_2_ shows less broadening than methane, possibly due to its linear geometry and different vibrational relaxation dynamics. Trapped nitrogen shows the least broadening, consistent with its smaller size. Interestingly, the free nitrogen peak appears broader than expected, possibly due to a fraction of nitrogen bound at intermediate positions in Cryptophane-A, i.e. interacting but not fully encapsulated. Such interaction may result in slightly red-shifted Raman signal relative to the free-state, but unresolved in the spectra. If present, this could mean nitrogen’s interaction with Cryptophane-A is somewhat underestimated.

Although Raman studies on cryptophane-A complexes with N_2_, CH_4_, and CO_2_ have not yet been reported, the observed red shift for encapsulated methane (26.8 cm^–1^) is in a good agreement with previously measured values for chloroform–Cryptophane-A complexes (28 cm^–1^),^23^ and exceeds that reported for methane clathrates at low temperatures (21.2 cm^–1^).^22^ This suggests a relatively strong host–guest interaction and a related weakening of the C–H bond. Similarly CO_2_ hydrates synthesized at high pressures and low temperatures show a Raman red shift of 8.5 cm^–1^, significantly smaller that cryptophane-A/CO_2_ complexes.^24^ However, binding constants and vibrational red shifts do not always correlate directly, and further validation through computational simulations would be necessary.^25^

It is important to note that simultaneous encapsulation of two guest molecules within a single cage is highly unlikely. Dual occupancy has not been observed for methane in solution and is even less likely for nitrogen due to its lower affinity and consequently shorter residence time within the Cryptophane-A cavity. In the case of CO_2_, its larger molecular dimension as well reduces the likelihood of co-occupancy within the cage.

## Conclusions

We employed Raman spectroscopy for the first time as a direct and effective method to demonstrate Cryptophane-A’s affinity for methane at room temperature, eliminating the need to extrapolate from low-temperature solution data. Moreover, we showed that the Cryptophane-A cavity is accessible not only to methane but also to nitrogen and carbon dioxide—two gases that have not been previously studied with this host. In all cases, gas molecules encapsulated within the cryptophane cage exhibited red-shifted Raman signals relative to their free-gas counterparts, consistent with positive host–guest interactions and van der Waals forces.

In addition, we quantified the relative selectivity of Cryptophane-A for these gases. While methane’s affinity is higher than that of nitrogen, the relatively modest selectivity factor of 2.5 suggests significant competitive binding between methane and nitrogen. This insight is particularly important, given that nitrogen is frequently used as a carrier gas in sensing applications, yet its interaction with cryptophanes has often been overlooked. As for CO_2_, the host exhibits approximately 1.5 times greater affinity for CO_2_ than for CH_4_. This direct measurement of CO_2_ capture in cryptophane-A cavities gives supports to an earlier indirect measurement done on cryptophane 111.^16^ Furthermore, our results reveal that molecular cages,^28^ such as cryptophanes, possess a stronger trapping capability for carbon dioxide under ambient conditions than has been reported in metal–organic frameworks (MOFs), which require high-pressure and low-temperature conditions.^26,27^

Our findings suggest that the cavity size of Cryptophane-A should be treated as an upper bound, enabling molecules with van der Waals volumes below ∼95 Å^3^ to interact with the host.^4^ This implies potential interferents such as water, ethanol, nitrogen, carbon dioxide, halomethanes, acetylene, ethane, xenon, argon, nitrogen oxides, ammonia, and others, raising significant implications for sensor design. In this context, Cryptophane-A-based sensors cannot be considered methane-specific, and pairing them with nonselective transduction methods (e.g., refractive index or mass sensors) is unreliable and unsuitable for environmental monitoring. Analytical specificity must be ensured using techniques such as IR, Raman, or mass spectrometry, particularly when analyzing unknown mixtures or when the relative affinities of the target gases are not well characterized. Nevertheless, cryptophanes, and Cryptophane-A in particular, remain valuable as preconcentration agents in combination with these techniques, offering potential for substantial improvements in detection limits.

Finally, it must be recognized that most reported sensor architectures employ cryptophane-doped polymer claddings rather than pure cryptophane. The polymer environment is expected to influence binding kinetics, selectivity, and the extent of analyte absorption, potentially modifying the behavior observed in pure crystals. Nevertheless, we hypothesize that our findings remain broadly valid for such claddings, even though composite layerss could not be directly verified with our Raman setup due to the low signal from the dilute cryptophane content in the material. To address this, we carried out Quartz Crystal Microbalance (QCM) measurements on cryptophane-doped styrene–acrylonitrile films (see Supporting Information, S3). Despite the intrinsic limitations of QCM, particularly its lack of molecular specificity, these measurements independently validated a higher affinity for methane over nitrogen and confirm cryptophane-A interaction with CO_2_. Crucially, they also leave open the possibility that CO_2_ may in fact bind more strongly than methane, underscoring the need for careful consideration of cross-sensitivity in future sensor designs.

Overall, Raman spectroscopy proved to be a highly specific and versatile tool for investigating gas encapsulation and host–guest interactions. The method can be extended to other environmentally relevant gases, such as hydrogen and nonprotic species that are challenging to study using NMR. Beyond Cryptophane-A, this approach is well-suited to examining other cage-like or porous host systems, including cyclodextrins, calixarenes, pillararenes, cucurbiturils, and materials such as MOFs (metal–organic frameworks), COFs (covalent organic frameworks), HOFs (hydrogen-bonded organic frameworks), and PIMs (polymers of intrinsic microporosity).

## Supplementary Material



## Notes

S1: NMR spectra of Cryptophane-A. S2: Raw data and error analysis. S3: QCM measurements and methods for gravimetric analysis of gas absorption in polystyrene-*co*-acrylonitrile–Cryptophane-A layers. (PDF)
