# Supplementary material for: Rethinking Cryptophane‑A for Methane Gas Sensing: Cross-Sensitivity to N2 and CO2 at Ambient Conditions
Source: Anal Chem. 2025 Sep 30;97(40):21793–8. doi: 10.1021/acs.analchem.5c03869 (PMC12529469; doi:10.1021/acs.analchem.5c03869)
Supplement: Supplementary file 1 [file ac5c03869_si_001.pdf]

Supporting Information

Rethinking Cryptophane-A for Methane Gas Sensing: Cross-Sensitivity to N_2 and CO_2 at Ambient Conditions

Sebastián Alberti[1], Thierry Brotin[2], Jana Jágerská[1]*

[1]Department of Physics and Technology, UiT The Arctic University of Norway, NO-9037, Tromsø, Norway.

[2]ENS de Lyon, CNRS, UMR 5182, Laboratoire de Chimie, Univ Lyon, Lyon 69342, France

KEYWORDS: Raman, cryptophane, methane, sensing, host-guest, molecular cages, preconcentration, enrichment.

Contents

S1. NMR measurement of synthesized cryptophanes 3

Figure S1.1. 1H NMR spectrum 3

Figure S1.2. 13C NMR spectrum........ 4

S2. Raw data and error analysis 5

Table S2.1. Raman peak positions and linewidths 5

Table S2.2. Fit goodness and Raman peak areas........ 5

S2.1 Statistical analysis and error propagation 6

S3. QCM measurement to assess CH_4 and CO_2 absorption in cryptophane-A doped polymer layers 7

Figure S3.1. Change of mass calculated using Sauerbrey equation for poly styrene-co-acrylonitrile layer (SAN) and cryptophane-A doped polymer layer (SAN-Crypt A) under exposure to different gases 8

S1. NMR measurement of synthesized cryptophanes

Cryptophane-A has been purified twice on silica gel and then recrystallized in a $CHCl_3$/EtOH mixture. The ^{1}H NMR and ^{13}C NMR spectra (Figures S1.1 and S1.2) show no significant impurities. We can estimate a purity above 99%.

Figure S1.1. ^{1}H NMR spectrum. Position of peaks and integration is displayed.

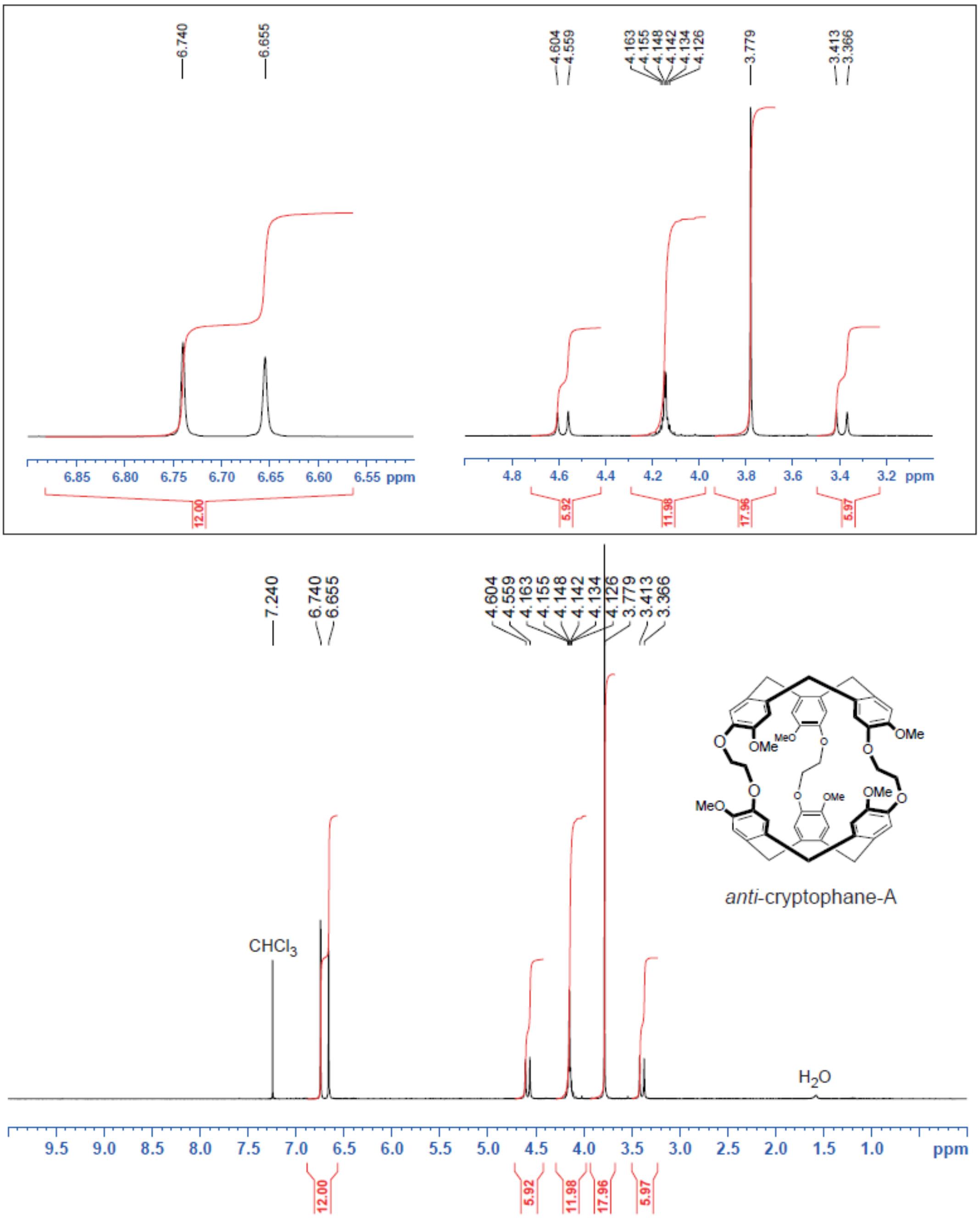

Figure S1.2. ^{13}C NMR spectrum.

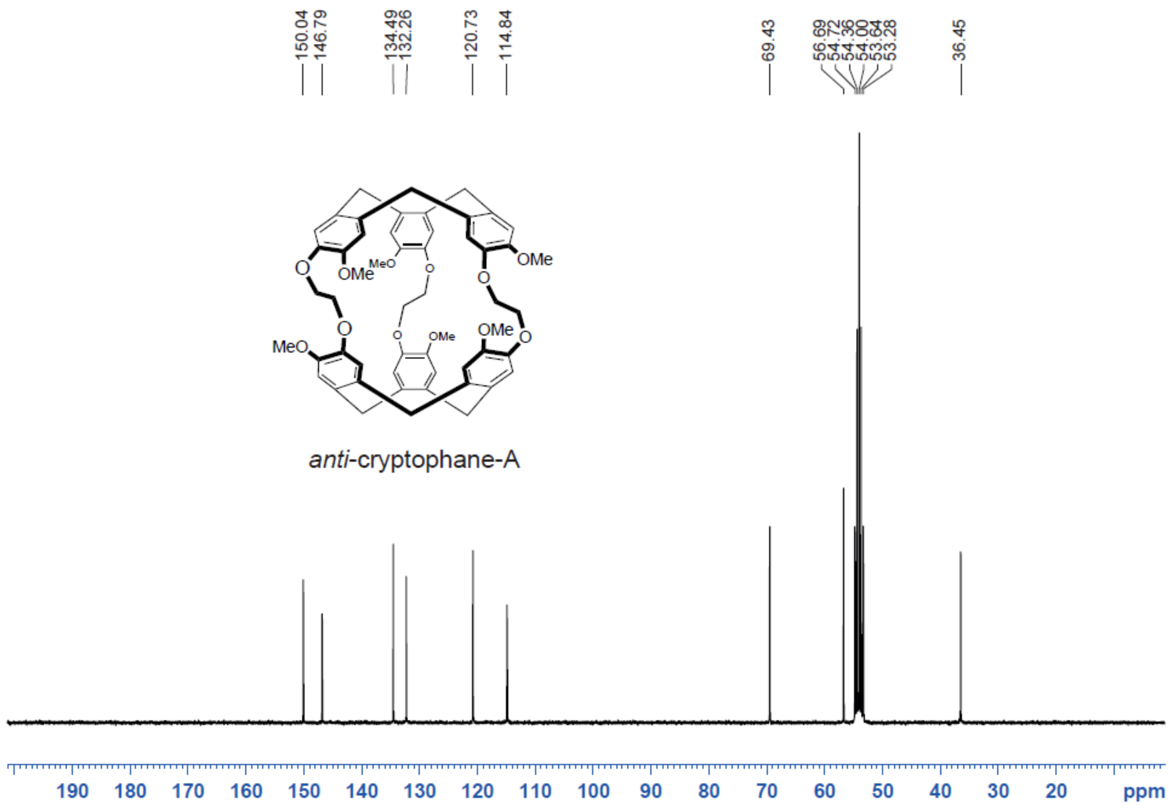

S2. Raw data and error analysis

A double Lorentzian fitting was done on each measured Raman spectrum after background subtraction. The free and trapped gas peak positions and peak widths are reported in Table S2.1. The peak areas are reported with their corresponding standard deviation and the goodness of the fit R^2 in Table S2.2. While the error on the peak position is a small fraction of a wavenumber while, the error on the peak area is considerably higher due to its sensitivity to the signal noise and baseline correction.

Table S2.1. Raman peak positions and linewidths for three independent Cryptophane-A-(CO_2/CH_4/N_2) measurements at different sample locations.

Gas	Free gas position (cm-1)	Free gas width (cm-1)	Trapped gas position (cm-1)	Trapped gas width (cm-1)	Redshift (cm-1)
CO_2 (2)	1391.6±0.2	1.5±1.0	1378.88±0.05	2.5±0.2	12.7±0.3
CO2 (1)	1389.8±0.2	1.7±0.5	1376.96±0.05	2.6±0.2	12.8±0.3
CO_2 (3)	1391.0±0.4	2.0±1.0	1378.5±0.1	2.8±0.3	12.5±0.5
CH_4 (2)	2922.1±0.1	1.8±0.3	2895.5±0.1	6.4±0.3	26.6±0.2
CH4 (1)	2920.9±0.1	1.4±0.2	2894.0±0.2	6.0±0.5	26.9±0.3
CH_4 (3)	2922.0±0.1	2.0±0.2	2895.0±0.2	6.3±0.5	27.0±0.3
N_2 (2)	2333.5±0.2	3.2±0.7	2322.38±0.05	2.4±0.2	11.1±0.3
N2 (1)	2331.8±0.1	2.8±0.3	2320.71±0.5	2.0±0.2	11.1±0.2
N_2 (3)	2332.9±0.1	3.1±0.4	2321.99±0.05	2.2±0.2	10.9±0.2

Table S2.2. Fit goodness and Raman peak areas for three independent Cryptophane-A-(CO_2/CH_4/N_2) measurements at different sample locations.

Area/ position	Location 1	Location 2	Location 3
CO_2	Area:<br>280966+-12701(trapped)<br>43467+-9794(free)<br><br>R^2:0.955	Area:<br>0.2898+- 0.0112<br>0.0319 +- 0.0085<br><br>R^2:0.965	Area:<br>0.318 +-0.029<br>0.054+-0.022<br><br>R^2: 0.919
CH_4	Area:<br>82073+-5780(trapped)<br>18645+-2250(free)<br><br>R^2:0.876	Area:<br>1.077+-0.033<br>0.1624+- 0.0158<br><br>R^2:0.941	Area:<br>80895+-5291<br>30866+- 2287<br><br>R^2:0.938
N_2	Area:<br>0.616+-0.032 (trapped)<br>0.498+-0.039(free)<br><br>R^2: 0.875	Area:<br>1.054+-0.046<br>0.357+-0.056<br><br>R^2:0.959	Area:<br>25976+-1256<br>18124+- 1562<br><br>R^2:0.954

S2.1 Statistical analysis and error propagation

For the error of the ratio between the areas A_e (A) and A_f (B) presented as error bars in Figure 4, as well as to calculate the selectivity values in Table 1, the following equation was used:

$$\frac{A}{B} = R \pm \sigma_R, \qquad \left(\frac{\sigma_R}{R}\right)^2 = \left(\frac{\sigma_A}{A}\right)^2 + \left(\frac{\sigma_B}{B}\right)^2$$

For the average selectivity reported in Table 1, the inverse-variance weighted average was used:

$$\textbf{Average} = \bar{x} \pm \sigma_{\bar{x}},$$

$$\bar{x} = \frac{\sum w_i x_i}{\sum w_i}, \ \sigma_{\bar{x}} = \sqrt{1/\sum w_i} \qquad \text{where } w_i = \frac{1}{(\sigma_i)^2},$$

S3. QCM measurement to assess CH_4 and CO_2 absorption in cryptophane-A doped polymer layers

Quartz Crystal Microbalance (QCM) measurements were performed to independently verify enrichment of methane and carbon dioxide in pure and cryptophane-A-doped (20% w/w) styrene-acrylonitrile (SAN) polymer films and evaluate their affinity relative to the carrier gas nitrogen.

QCM-I from MicroVacuum with impedance analysis and temperature control was employed as the measurement instrument, while gas-tight with PEEK tubing and components were used to prevent any gas sample contamination or concentration variation uncertainty. New quartz crystal sensors with Ti/Au electrodes were spin coated at 1000 rpm with a 4% Polyacrylonitrile solution in tetrachloroethane with and without Cryptophane-A (doping was 20% in weight). The samples were dried at 50ºC for two days under vacuum. The layer thickness and refractive index was measured with a spectroscopic ellipsometer (Semilab). No significant changes of the refractive index between doped and undoped layers suggest that no major changes occurred in the polymer layer structure. Prior to the measurement, a baseline was registered flowing 100% nitrogen at 25 ºC. Next, 100% methane and 100% carbon dioxide were measured in two sequential measurements, until stable signal was collected. Signal stabilization occurred within seconds for carbon dioxide and within a few minutes for methane, i.e. markedly faster than in Raman measurements on crystals, where stabilization required up to two hours. This rapid response can be attributed to the nanometric thickness of the QCM layer and the porous nature of the amorphous polymer matrix. In the last step, the nitrogen flow was restored, to retrieve the baseline signal.

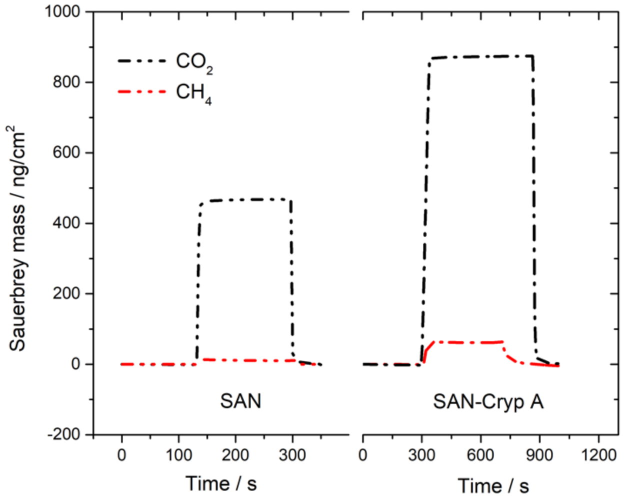

Figure S3.1. Change of mass calculated using Sauerbrey equation for poly styrene-co-acrylonitrile layer (SAN) and cryptophane-A doped polymer layer (SAN-Crypt A) under exposure to different gases. The response to both CH_4 and CO_2 when N_2 is used as the reference gas is substantially enhanced when the polymer layer is doped.

The change of the Sauerbrey mass is presented in Figure S3.1. The undoped layer with a thickness of 850 nm increased its mass by 475 ng/cm^2 and 10 ng/cm^2 when flowing CO_2 and methane, respectively. The doped layer with the same thickness showed an additional increase of 410 ng/cm^2 for CO_2 and an increase of 50 ng /cm^2 for methane when compared to undoped layers. The response to nitrogen could not be quantified, as nitrogen was the carrier gas and served as the baseline.

The result clearly supports the stronger binding for methane than nitrogen: Methane gas has namely a lower molecular mass than nitrogen and still shows an increase in the mass under methane flow when compared to nitrogen. These can only be attributed to an increased abundance of gas molecules in the layer, achieved through more cryptophane cages occupied with methane then they were previously occupied with nitrogen. This agrees with the hypothesis

of stronger affinity to methane, resulting in stronger binding and longer dwelling time for methane than under nitrogen flow. This is consistent with the result obtained using Raman spectroscopy.

Interpretation of the data for carbon dioxide, on the other hand, should be taken carefully. Due to its higher molecular mass, the additional increase of the mass does not automatically imply that more cages are occupied by carbon dioxide than by nitrogen despite an eight-fold change in the Sauerbrey mass when compared to methane. Nevertheless, a limit cases can be analysed: negligible affinity for nitrogen. This assumption would imply a CO_2/CH_4 selectivity of 2.9. This value is the upper selectivity limit, assuming cryptophane is not affected by the polymer matrix and vice versa. As shown in Raman measurements nitrogen interaction is not negligible and therefore the selectivity is lower than 2.9.

To quantify the affinity to methane and carbon dioxide from the QCM measurement, it would be desirable to use a non-interacting gas as a carrier gas, i.e., big gas molecules unable to enter the cavity such as SF_6 or the smallest gas, He, to minimize van der Waals forces. Alternatively, a QCM vacuum/high pressure module could be used, completely removing the need for a carrier gas. Additionally, working with less interacting polymers such as perfluorinated polymers would decrease the uncertainty due to molecular adsorption in the polymer layer.
